# Performance Enhancement of Pharmacokinetic Diffuse Fluorescence Tomography by Use of Adaptive Extended Kalman Filtering

**DOI:** 10.1155/2015/739459

**Published:** 2015-05-19

**Authors:** Xin Wang, Linhui Wu, Xi Yi, Yanqi Zhang, Limin Zhang, Huijuan Zhao, Feng Gao

**Affiliations:** ^1^College of Precision Instrument and Optoelectronics Engineering, Tianjin University, Tianjin 300072, China; ^2^Tianjin Key Laboratory of Biomedical Detecting Techniques and Instruments, Tianjin 300072, China

## Abstract

Due to both the physiological and morphological differences in the vascularization between healthy and diseased tissues, pharmacokinetic diffuse fluorescence tomography (DFT) can provide contrast-enhanced and comprehensive information for tumor diagnosis and staging. In this regime, the extended Kalman filtering (EKF) based method shows numerous advantages including accurate modeling, online estimation of multiparameters, and universal applicability to any optical fluorophore. Nevertheless the performance of the conventional EKF highly hinges on the exact and inaccessible prior knowledge about the initial values. To address the above issues, an adaptive-EKF scheme is proposed based on a two-compartmental model for the enhancement, which utilizes a variable forgetting-factor to compensate the inaccuracy of the initial states and emphasize the effect of the current data. It is demonstrated using two-dimensional simulative investigations on a circular domain that the proposed adaptive-EKF can obtain preferable estimation of the pharmacokinetic-rates to the conventional-EKF and the enhanced-EKF in terms of quantitativeness, noise robustness, and initialization independence. Further three-dimensional numerical experiments on a digital mouse model validate the efficacy of the method as applied in realistic biological systems.

## 1. Introduction

In diffuse fluorescence tomography (DFT) regime, pharmacokinetic imaging means a dynamic modality that ultimately acquires the spatially varying pharmacokinetic parameters of administrated fluorescent agent in tissue [1]. Among all the commercially available fluorescent agents, only indocyanine green (ICG) is approved for human use by the U.S. Food and Drug Administration. As a blood pooling agent, ICG has evidently distinct delivery behavior between cancerous tissue and normal tissue due to the proliferation of the “leaky” angiogenetic microvessels [2]. Therefore, the pharmacokinetic-DFT of ICG potentially provides contrast- and specificity-enhanced information for tumor diagnosis, malignancy staging, treatment monitoring, and drug-delivery assessment, as compared to the static modality that only discloses the temporally averaged fluorophore concentration image [3].

The current image reconstruction methods for the pharmacokinetic-DFT can be categorized into explicit and implicit schemes, both combining the DFT principle with pharmacokinetic analysis. In the explicit scheme the pharmacokinetic images are calculated in a voxel-by-voxel fashion by fitting a kinetic model to the reconstructed temporal sequence of the fluorophore concentration [4], while in the implicit scheme the pharmacokinetic images are directly reconstructed as a whole by incorporating a deterministic kinetic model to the inversion procedure [5] or by expressing the kinetics-to-measurement map in the extended Kalman filtering (EKF) procedure [6]. Although it is demonstrated that the implicit scheme substantially improves accuracy and robustness in pharmacokinetic parameter estimation, the methodology is theoretically complex and computationally costly due to the further degradation of ill-posedness and increased unknowns in the inversion, thus limiting its application to two-dimensional (2D) scenarios [5, 6]. In this sense, the explicit scheme is universally applicable and relatively easy-to-implement but needs to be enhanced for its performance and robustness in both the concentration reconstruction and the parameter estimation.

Compartmental modeling is a well-established approach to the pharmacokinetic analysis. This method describes the concentration dynamics as a result of fluorophore exchange among kinetically distinct compartments using a set of coupled ordinary differential equations (ODEs) with its coefficients representing the exchange rates, referred to as the pharmacokinetic-rates. A biexponential-curve-fitting method based on the two-compartment model has been proposed to demonstrate the feasibility of the ICG pharmacokinetics in tumor diagnosis [4, 7–9]. Alacam and Yazici have addressed an EKF framework for estimating the pharmacokinetic-rates of highly nonlinear nature and validated the sufficiency of the two-compartment model in describing ICG kinetics [3]. Furthermore, an EKF study using the two-compartment model has indicated the superiority of the pharmacokinetic-rate images to the bulk rates of entire breast in cancer diagnosis [10]. In comparison with the curve-fitting techniques, the EKF-based method has a number of advantages: (1) the EKF regards the ICG dynamics as an evolutionary stochastic process and therefore provides a better fit to the fluorophore metabolism than the exponential-curve-based model; (2) the EKF can readily accommodate spatiotemporal priors of the kinetic parameters; (3) the EKF can achieve real-time estimation of a complete set of the pharmacokinetic parameters including pharmacokinetic-rates and concentrations in different compartments; and (4) the method is universally applicable to any optical fluorophore [3, 6].

However, the performance of the conventional-EKF highly hinges on the exact prior knowledge about the initial states, that is, the expectations and covariances of the compartmental concentrations and pharmacokinetic parameters, which are always inaccessible in practice. The inappropriate initialization can cause the degeneration even the divergence of the EKF [11–13]. Ozbek et al. have presented an enhanced-EKF scheme to improve the accuracy of the EKF analysis [14, 15], where a forgetting-factor is introduced to compensate the inaccuracy of the initial states and to emphasize the effect of the current data. This strategy in principle better stabilizes the online estimation compared with the conventional-EKF [16]. Nevertheless, a constant forgetting-factor is intrinsically suboptimal, for example, the larger forgetting-factor results in a larger Kalman gain means that the filter can quickly adapt to a new situation but also that it is sensitive to random errors. By nature, adopting a variable forgetting-factor that adaptively balances between the convergence and robustness will further improve the performance of the EKF.

We herein propose an adaptive-EKF for DFT-based pharmacokinetic imaging, where the forgetting-factor is updated at each recursive stage, by contrasting the calculated innovation covariance and the estimated one. For the methodology to be universally applicable, the algorithm is implemented within the framework of the explicit scheme that firstly reconstructs the time-course of ICG concentration with the conventional-DFT and then accordingly estimates the pharmacokinetic-rate images using the adaptive-EKF approach for the two-compartment model. Simulation results of a two-dimensional circular model suggest that the proposed adaptive-EKF can obtain preferable pharmacokinetic-rate images to both the conventional- and enhanced-EKF in terms of quantitativeness, noise robustness, and initialization independence. Further numerical experiments on a three-dimensional (3D) digital mouse model validate the feasibility and efficacy of the method as applied in 3D complex biological systems.

## 2. Theory

### 2.1. Two-Compartment Model

**Figure 1:**
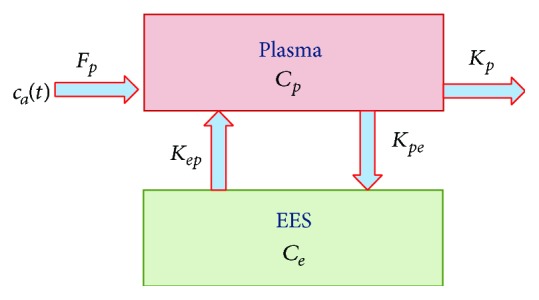
Two-compartmental model of ICG pharmacokinetics. *C*_*p*_ and *C*_*e*_ denote the tissue concentrations of ICG in plasma and EES, that is, the numbers of ICG molecules in plasma and EES relative to the total tissue volume, respectively, *K*_*pe*_, *K*_*ep*_, and *K*_*p*_ are pharmacokinetic-rates describing the ICG leakage into and the drainage out of the EES, as well as the ICG elimination from the body through circulatory system, respectively, *c*_*a*_(*t*) is the arterial input function (AIF), and *F*_*p*_ is volume flow.

Compartmental modeling assumes that a biological system is conceptually (not geometrically) divided into a series of compartments, each representing a well-mixed space of similar tissues within which the fluorophore is uniformly distributed and its concentration changes as a result of the agent exchange among the compartments. Mathematically, the kinetic changes of the compartmental concentrations are governed by a collection of coupled ODEs, ultimately resting on the principle of mass conservation [17–20]. In the two-compartment model, tissue is composed of plasma and the extracellular-extravascular space (EES), as shown in Figure 1, where *C*_*p*_(**r**, *t*) and *C*_*e*_(**r**, *t*) denote the tissue concentrations of ICG in plasma and EES, that is, the numbers of ICG molecules in the two compartments relative to the total tissue volume, respectively; *K*_*pe*_(**r**), *K*_*ep*_(**r**), and *K*_*p*_(**r**) are pharmacokinetic-rates describing the ICG leakage into and the drainage out of the EES, as well as the ICG elimination from the body through circulatory system, respectively; *c*_*a*_(*t*) is the arterial input function (AIF), and *F*_*p*_ is volume flow [17]. With the above notations, the two-compartment model of ICG is described by the following ODEs set:

(1) dCer,tdtdCpr,tdt=−KeprKperKepr−Kper−Kpr ·Cer,tCpr,t+0Fpcat.

It is important to distinguish the tissue compartmental concentrations, *C*_*χ*_(**r**, *t*) (*χ* ∈ {*p*, *e*}), used in the above ODE from the local compartmental concentrations, *c*_*χ*_(**r**, *t*) = *C*_*χ*_(**r**, *t*)/*v*_*χ*_, with *v*_*p*_ and *v*_*e*_ being the fractions of plasma and EES volumes, respectively [17, 18]. The latter is essentially the ratio of the ICG molecule number in plasma or EES compartment to its fractional volume and has been inappropriately used in the previous works [3, 6, 10, 21].

With the explicit scheme of dynamic DFT, the time course of the total ICG concentration in tissue, *C*(**r**, *t*) = *C*_*e*_(**r**, *t*) + *C*_*p*_(**r**, *t*), is tomographically reconstructed at discrete time instances, *C*(**r**, *k*) = *C*(**r**, *k*Δ*T*), (*k* = 1,2,…, *K*), where Δ*T* is the sampling period. Here we only consider the permeability of ICG in the EKF process after agent administration. In order to achieve the joint estimation of the pharmacokinetic-rates and the ICG tissue concentrations within the EKF framework, a dynamic model of the parameter vector is additionally appended to the two-compartment model to construct a discrete nonlinear state-space model as follows [3, 6, 17–20]:

(2) $$\begin{array}{c} \left[\begin{array}{c} C_{ep}\left(r,k+1\right)\\ \theta \left(r,k+1\right) \end{array}\right]=\left[\begin{array}{c} Κ\left(\theta \left(r,k\right)\right)C_{ep}\left(r,k\right)\\ \theta \left(r,k\right) \end{array}\right]+\left[\begin{array}{c} \omega \left(r,k\right)\\ \varsigma \left(r,k\right) \end{array}\right]\\ C\left(r,k\right)=EC_{ep}\left(r,k\right)+\eta \left(r,k\right), \end{array}$$

where $C_{ep}\left(r,k\right)={\left[\begin{array}{cc} C_{e}\left(r,k\right) & C_{p}\left(r,k\right) \end{array}\right]}^{T}$ is the vector representing the compartmental concentrations with *C*_*e*_(**r**, *k*) = *C*_*e*_(**r**, *k*Δ*T*) and *C*_*p*_(**r**, *k*) = *C*_*p*_(**r**, *k*Δ*T*); $E=\left[\begin{array}{cc} 1 & 1 \end{array}\right]$; **ω**(**r**, *k*), **ς**(**r**, *k*), and *η*(**r**, *k*) are independent zero-mean Gaussian white noise processes with the covariance matrices **Q** and **Z** and the variance *R*, referred to as the state driving noise vector, parameter driving noise vector, and observation noise, respectively; $\theta \left(r,k\right)={\left[\begin{array}{cccc} \tau_{11}\left(r,k\right) & \tau_{12}\left(r,k\right) & \tau_{21}\left(r,k\right) & \tau_{22}\left(r,k\right) \end{array}\right]}^{T}$ is the parameter estimation at time *k*Δ*T*; **K**(***θ***) is the pharmacokinetic-rates-related system matrix for the discrete time two-compartment model as follows:

(3) Kθ=τ11rτ12rτ21rτ22r=exp⁡−KeprKperKepr−Kper+KprΔT.

### 2.2. The General Framework of EKF

Let ${\hat{C}}_{ep}\left(r,k\right)$ and $\hat{\theta }\left(r,k\right)$ be the estimation of the compartmental concentrations **C**_*ep*_(**r**, *k*) and the parameters ***θ***(**r**, *k*) at the *k*th step, respectively, let ${\hat{C}}_{ep}\left(r,k|k-1\right)$ and $\hat{\theta }\left(r,k|k-1\right)$ be the one-step ahead prediction of **C**_*ep*_(**r**, *k*) and ***θ***(**r**, *k*), respectively. The general framework of an EKF for the two-compartment model is summarized as a recursive procedure of “Prediction-Gain-Update” [4, 6, 13–16].

*(1) Prediction. *Consider

(4) $$\begin{array}{c} \left[\begin{array}{c} {\hat{C}}_{ep}\left(r,k\;|\;k-1\right)\\ \hat{\theta }\left(r,k\;|\;k-1\right) \end{array}\right]=\left[\begin{array}{c} K\left(\hat{\theta }\left(r,k-1\right)\right){\hat{C}}_{ep}\left(r,k-1\right)\\ \hat{\theta }\left(r,k-1\right) \end{array}\right]. \end{array}$$

*(2) Kalman Gain. *Consider

(5) Pr,k ∣ k−1=λr,kJr,k−1Pr,k−1JTr,k−1 +Q00Z,Gr,k=Pr,k ∣ k−1ΛTDr,kPr,k=I−Gr,kΛPr,k ∣ k−1,

where $\Lambda =\left[\begin{array}{cccccc} 1 & 1 & 0 & 0 & 0 & 0 \end{array}\right]$, **J**(**r**, *k* − 1) is the Jacobian matrix of the nonlinear EKF system, *D*(**r**, *k*) = Λ**P**(**r**, *k*∣*k* − 1)Λ^*T*^ + *R* is referred to as the calculated covariance since it equals the covariance of the innovation sequence $d(r,k)=C(r,k)-E{\hat{C}}_{ep}(r,k|k-1)$ as the model is accurate [13], **I** is an identity matrix, and *λ* is a forgetting-factor. To obtain the Kalman gain, **G**(**r**, *k*), the one-step ahead prediction of the error-covariance matrix, **P**(**r**, *k*∣*k* − 1), is firstly calculated in terms of the estimated error-covariance matrix at the previous step, **P**(**r**, *k* − 1), and then is updated to reach its estimation at the current step, **P**(**r**, *k*), for the next recursion. According to the forgetting-factor *λ*, the EKF can be classified as the conventional (*λ* = 1) [4, 6, 10] or the enhanced (*λ* is a constant larger than 1) [14, 15]. 

*(3) Update. *Consider

(6) $$\begin{array}{c} \left[\begin{array}{c} {\hat{C}}_{ep}\left(r,k\right)\\ \hat{\theta }\left(r,k\right) \end{array}\right]=\left[\begin{array}{c} {\hat{C}}_{ep}\left(r,k\;|\;k-1\right)\\ \hat{\theta }\left(r,k\;|\;k-1\right) \end{array}\right]+d\left(r,k\right)G\left(r,k\right). \end{array}$$

Prior to the recursive process, the EKF can be initialized theoretically for the state, that is, expectations of ICG concentrations and ***θ*** and the error covariance matrix, that is, $P_{0}=[\begin{array}{cc} \text{Cov}\left(C_{ep}\left(r,0\right)\right) & 0\\ 0 & \text{Cov}\left(\theta \left(r,0\right)\right) \end{array}]$. In practice, $\hat{\theta }\left(r,0\right)$ is always experientially chosen as the physiologically relevant values. ${\hat{C}}_{ep}\left(r,0\right)$ can be calculated from the initial quantities of intravenous injection. It is emphasized that the filter may degenerate or even diverge with the improper initialization [11–13].

### 2.3. Adaptive-EKF with a Variable Forgetting-Factor

Theoretically, a linear filter can be considered an optimal one as its innovation sequence is white. According to this criterion, however, the innovation sequence of the EKF is not white due to the linearization error. Nevertheless, the performance of the EKF might still be improved on a condition of temporal independence of the innovation; that is, the autocovariance of the innovation sequence is zero. To approach such a condition, a temporally varying forgetting-factor *λ*(**r**, *k*) is introduced as [16]

(7) $$\begin{array}{c} \lambda \left(r,k\right)=\max\left\{1,\log\left(\frac{\overset{-}{D}\left(r,k\right)}{D\left(r,k\right)}\right)\right\}, \end{array}$$

where $\overset{-}{D}(r,k)$ is referred to as the estimated innovation covariance:

(8) $$\begin{array}{c} \overset{-}{D}\left(r,k\right)=\left\{\begin{array}{cc} d{\left(r,k\right)}^{2}, & k<W\\ \left(\frac{1}{W}\right)\overset{k}{\underset{i=k-W+1}{\sum }}d{\left(r,i\right)}^{2}, & k\geq W. \end{array}\right. \end{array}$$

The essence of the above equation is to estimate the real innovation covariance by averaging inside a moving estimation window of size *W* [22]. The windowing strategy in the calculation helps balance between the confidences in the “old” observation data and the current data. No optimal criterion is thus far found for selection of the window width *W* and some care must be taken with this issue. In principle, the filtering might fail to mitigate the negative effects of the “old” observation data with a large *W* and, on the contrary, would be adversely affected by sudden noise in the new data as *W* is too small.

It is difficult in practice to acquire the exact nonlinear stochastic equations of the pharmacokinetic system, due to the unavailability of the noise characteristics. With inaccurate initialization of the noise characteristics, the calculated innovation covariance *D*(**r**, *k*) may be lower than the estimated one $\overset{-}{D}(r,k)$, leading to a forgetting-factor greater than 1, that is, *λ*(**r**, *k*) > 1. The predicted error covariance **P**(**r**, *k*∣*k* − 1) is then amplified by the forgetting-factor to ameliorate the inexact modeling and emphasize the role of the current data. On the other hand, it must be ensured that *λ*(**r**, *k*) ≥ 1 to stabilize the filtering process. Since a sharp variation of the ratio of the estimated innovation covariance $\overset{-}{D}(r,k)$ to the calculated one *D*(**r**, *k*) may occur during the rapidly varying early phase of the ICG kinetic process after injection, due to the inappropriate initialization of the filter or some unaccounted perturbation [2], a logarithmic ratio value of $\overset{-}{D}(r,k)$ to *D*(**r**, *k*) is used to avoid the rapid local convergence under a large forgetting-factor.

## 3. Simulative Investigations

Using the pharmacokinetic-rates listed in Table 1, the time-course of the ICG concentration, *C*(**r**, *k*), is calculated based on the two-compartment kinetic model, that is, (2), that are driven by the zero-mean Gaussian noises, **ω**(**r**, *k*), with signal-to-noise ratio (SNR), SNR_*ω*_, respectively. The pharmacokinetic-rates are assumed to be nearly constant at the clinical-pathologic stage as the measurement is performed, a high SNR of SNR_*ς*_ = 60 dB (0.1%) is used to drive the parameter vector ***θ***(**r**, *k*) in the simulations. In the calculation, we set the initial concentration of ICG to *C*(0) = 1.0 *μ*M and assume that all the ICG is in the plasma at *t* = 0 s.

With the time-course of the ICG concentration, *C*(**r**, *k*), we can accordingly calculate the time-varying fluorephore absorption coefficient, *μ*_af_(**r**, *k*), in terms of a linear relationship of *μ*_af_(**r**, *k*) = ln⁡10*εC*(**r**, *k*), where *ε*  ( = 0.013 mm^−1^ *μ*M^−1^) is the extinction coefficient of ICG, and finally obtain the boundary flux by solving the coupled diffusion equations using the finite element method, as the simulated data for the reconstruction [23–27]. The investigations herein rely on combination of the multichannel photon-counting DFT system custom-made in our lab [21] and the widely-adopted normalized Born formulation for DFT reconstruction [23, 27]. This means that both the excitation and emission SNRs, that is, SNR_*x*_ and SNR_*m*_, can achieve reasonably high levels of above 30 dB and 20 dB, respectively, and an optically homogeneous background can be used in the forward calculation.

To further obtain the estimated pharmacokinetic parameters ${\hat{C}}_{ep}\left(r,k\right)$ and $\hat{\theta }\left(r,k\right)$, the DFT-reconstructed ICG concentration, *C*′(**r**, *k*), is analyzed by conventional-, enhanced-, and adaptive-EKF procedures, respectively. The constant forgetting-factor of the enhanced-EKF is set to be 1.1 in the simulations according to [15], while the adaptive-EKF adopts a 7-length (*W* = 7) window. The noise covariance matrices of the state and parameter driving noise vectors in the filter are set to **Q** = 10^−8^ *C*(0)**I** and **Z** = 10^−5^ ***θ***(0)**I**, and the variance *R* of the observation noise is dependent on the additive noise levels in the measurements [6].

### 3.1. 2D Circular Phantom

**Figure 2:**
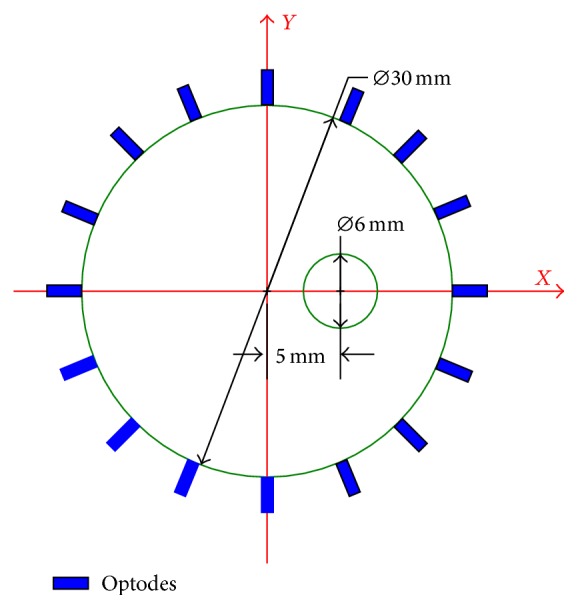
Sketch of the 2D phantom model and source-detector configuration.

A circular phantom with 30 mm diameter is used that embeds a circular tumor-emulating region of 6 mm diameter, as shown in Figure 2. Its absorption coefficient and reduced scattering coefficient are set to *μ*_*a*_ = 0.035 mm^−1^ and *μ*_*s*_′ = 1.0 mm^−1^ for both the excitation and emission wavelengths. The domain is discredited by a finite element mesh with 721 nodes and 1350 elements (triangles) for both the forward and inverse calculations and sampled by 16 coaxial source-detector optodes that are placed around the phantom with equal spacing. To acquire a complete dataset for the 16 × 16 source-detector combinations, the 16 detectors collect the photons in parallel as 16 sources illuminate the surface in serial. The measurement is repeatedly conducted for 720 s (12 minutes) at a sampling period of Δ*T* = 10 s, generating 72 datasets of the time-course for the pharmacokinetic estimation.

For the pharmacokinetic-rates, *K*_*pe*_ and *K*_*ep*_ are set to represent three target-to-background contrasts in agreement with the different tumor pathological staging, while *K*_*p*_ is assumed to be homogeneous and known throughout the simulations, as shown in Table 1.

**Figure 3:**
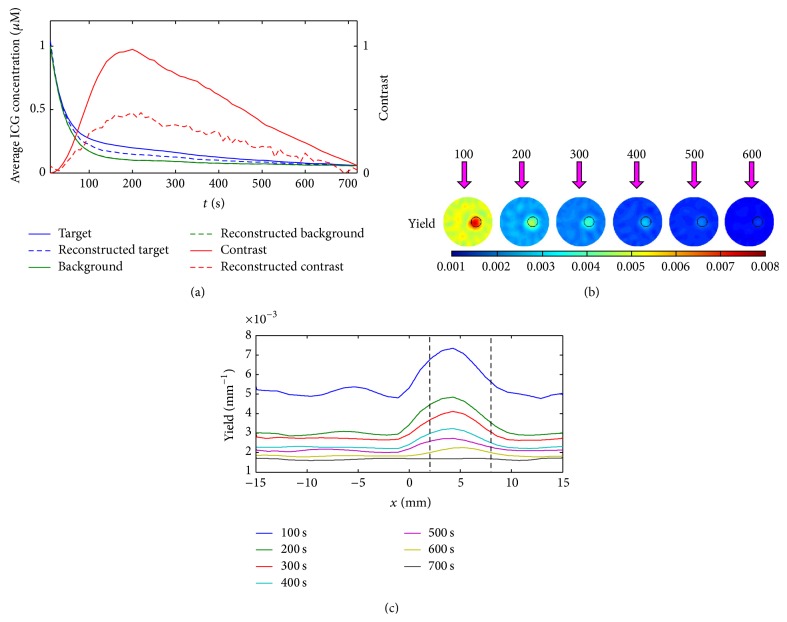
The time-course and contrast of the model-simulated and DFT-reconstructed average ICG concentrations in the background and target areas, *C* and *C*′ (a), as well as the interim yield-images (b) and their X-profiles (c) at *t* = 100, 200, 300, 400, 500, 600 s. The calculations are performed for the pharmacokinetic-rates of contrast = 2 from the simulated data with SNR_*ω*_ = 40 dB, SNR_*ς*_ = 60 dB, SNR_*x*_ = 55 dB, and SNR_*m*_ = 45 dB. The black circles indicate the ideal location and size of the targets.

To demonstrate the above dynamic DFT procedure, Figure 3 illustrates the time-course of the model-simulated and DFT-reconstructed ICG average concentrations in the background and target regions, *C*(*k*) and *C*′(*k*), as well as the time-course of the true and reconstructed contrasts, for the scenario of contrast = 2 in Table 1. Here we define the contrast as the ratio of the average target variation to the average background [28]. For the reference, the interim DFT-reconstructed yield-images and their X-profiles are also given at six time instants of 100 s to 600 s, which are generated from the simulated time-course of ICG concentration with SNR_*ω*_ = 40 dB, SNR_*ς*_ = 60 dB, SNR_*x*_ = 55 dB, and SNR_*m*_ = 45 dB. It is found that the reconstructed concentration features an increased underestimation with increased true contrast during the kinetic process: an adversity originates from the underestimation of high yield contrasts in DFT-reconstruction.

#### 3.1.1. Quantitativeness

**Figure 4:**
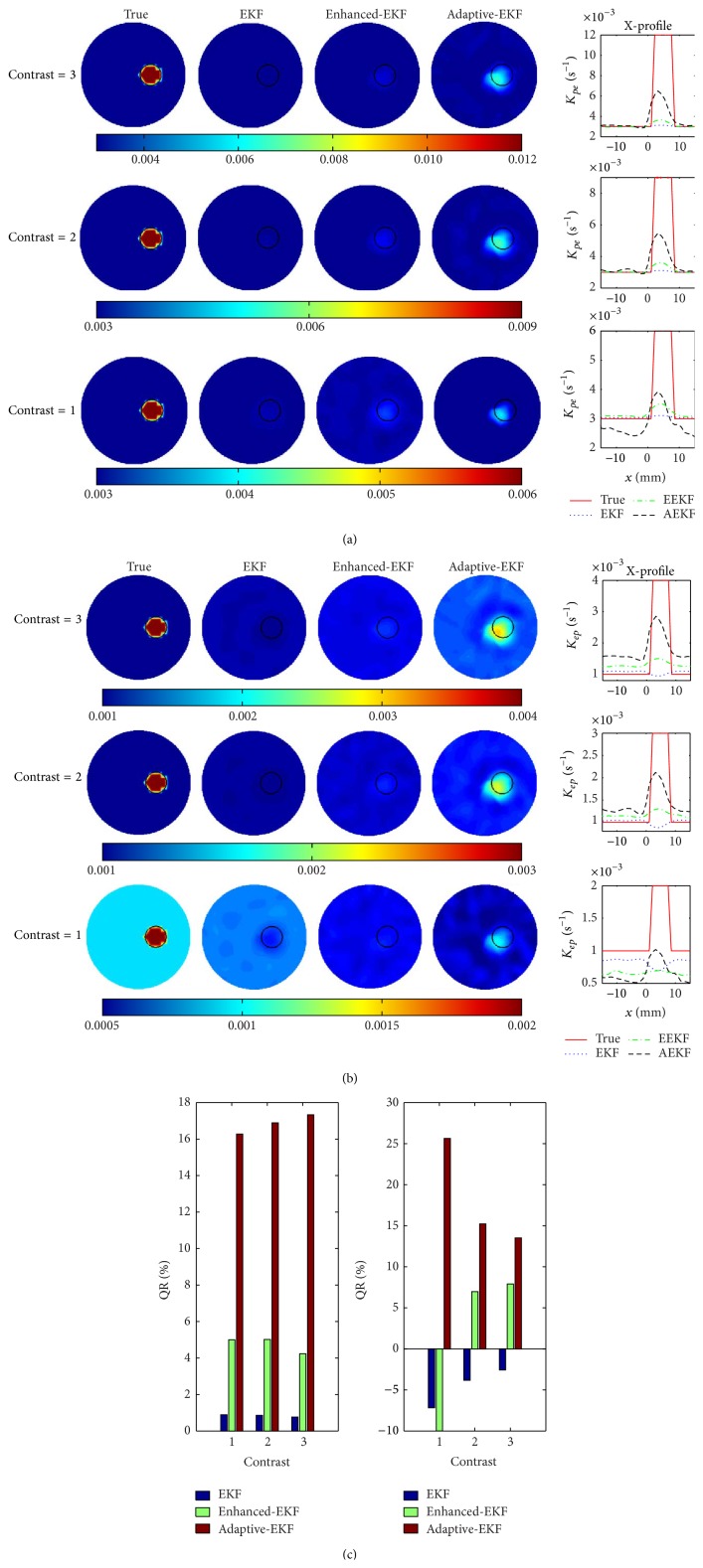
The estimated results of pharmacokinetic-rates: (a) images and X-profiles of *K*_*pe*_ with contrast = 3 (top), contrast = 2 (middle), and contrast = 1 (bottom), (b) images and X-profiles of *K*_*ep*_ with contrast = 3 (top), contrast = 2 (middle), and contrast = 1 (bottom), and (c) the quantitativeness ratio (QR) of *K*_*pe*_ (left) and *K*_*ep*_ (right).

The images of the pharmacokinetic-rates, *K*_*pe*_ and *K*_*ep*_, estimated by the conventional-, enhanced-, and adaptive-EKFs, for the three sets of the true target-to-background contrasts in Table 1, are illustrated in Figures 4(a) and 4(b), respectively. In the filtering process, the variance of the observation noise is chosen as *R* = 3 × 10^−4^ *C*(0), and the initial pharmacokinetic-rates are set to be same for the three filters, that is, *K*_*pe*_ = 0.003 s^−1^ and *K*_*ep*_ = 0.001 s^−1^.

**Figure 5:**
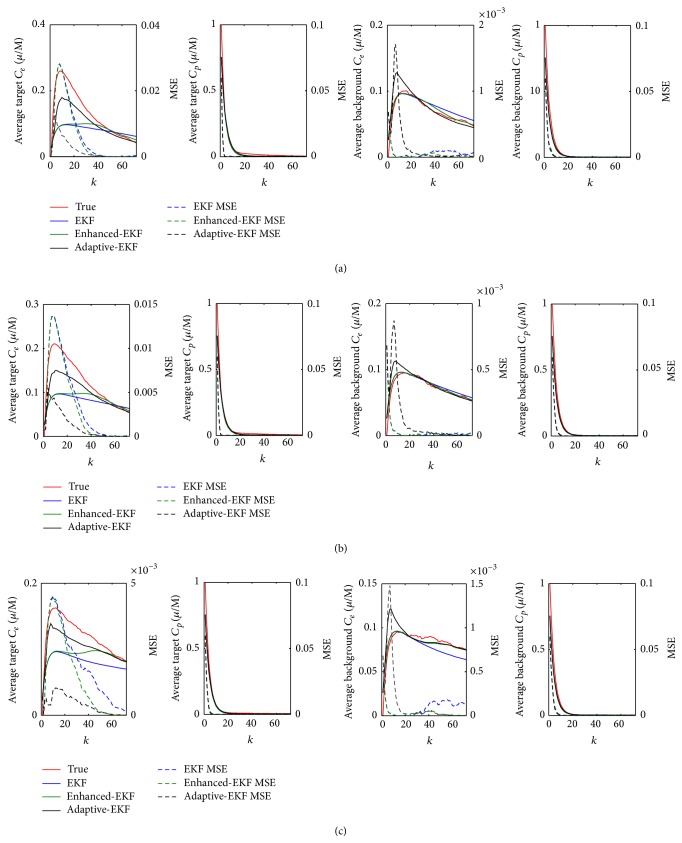
The estimated average time-course of the compartmental concentration and their MSEs in the target and background areas: (a) contrast = 3, (b) contrast = 2, and (c) contrast = 1.

A good agreement between the true and the estimated images is observed in terms of the localization and size of the target. It can be found in terms of the X-profiles that the adaptive-EKF outperforms the conventional-EKF and enhanced-EKF in estimation accuracy. For quantitative assessment of the method, the quantitativeness ratio (QR), defined as the ratio of the estimated contrast to the true one of a pharmacokinetic-rate, as shown in Figure 4(c). The much higher QRs of the *K*_*pe*_- and *K*_*ep*_-images achieved by the adaptive-EKF exhibit the enhanced ability of the method to dynamically compensate the initial inaccuracies. This feature is further quantified with the time-course of the mean square error (MSE) between the estimated and true compartmental concentration images, defined as

(9) $$\begin{array}{c} \text{MS}{\text{E}}_{\chi }\left(k\right)=\left(\frac{1}{N}\right)\overset{N}{\underset{n=1}{\sum }}{\left[{\hat{C}}_{\chi }\left(n,k\right)-C_{\chi }\left(n,k\right)\right]}^{2}, \end{array}$$

where *χ* ∈ {*e*, *p*}; *N* is the number of the nodes in the region of interest; ${\hat{C}}_{\chi }\left(n,k\right)$ and *C*_*χ*_(*n*, *k*) are the estimated and the true compartmental concentrations at the *i*th node, respectively.  Figure 5 shows the estimated average time-course of ICG compartmental concentrations and their MSEs in the target and background areas. It is observed, for the three pharmacokinetic contrasts, that the average *C*_*e*_ in the target area estimated by adaptive-EKF converges faster to the true value and realizes a smaller MSE than the conventional and the enhanced ones. For *C*_*p*_ estimation, the three filters exhibit almost the same performance according to the figure.

#### 3.1.2. Noise Robustness

To evaluate the noise robustness of the three filters, the estimations are conducted for the pharmacokinetic contrast of 2, with different levels of the state driving noise and measurement noise, as shown in Table 2. The initial pharmacokinetic-rates are set to be same for the three filters, that is, *K*_*pe*_ = 0.003 s^−1^ and *K*_*ep*_ = 0.001 s^−1^.

**Figure 6:**
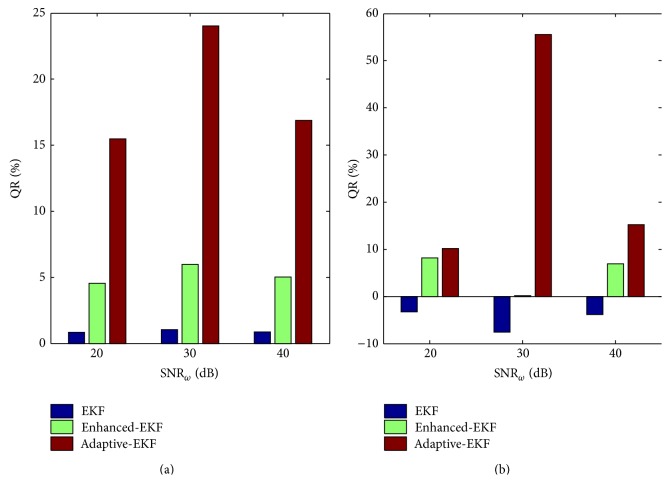
The estimated QRs of *K*_*pe*_ (a) and *K*_*ep*_ (b) with different state driving noise levels.

Firstly, to compare the robustness of the three filters to the state driving noise, the data is generated with SNR_*ω*_ = 20 dB, 30 dB, and 40 dB, by fixing reasonably low levels of the measurement noise: SNR_*x*_ = 55 dB and SNR_*m*_ = 45 dB, that is, Case 1, Case 2, and Case 3 in Table 2. A fixed observation noise variance of *R* = 3 × 10^−4^ *C*(0) is used in the filtering process.  Figure 6 contrasts the resultant QRs of *K*_*pe*_ and *K*_*ep*_ for the three cases. It is seen that the QRs achieved by the adaptive-EKF are much higher than the other two schemes.

**Figure 7:**
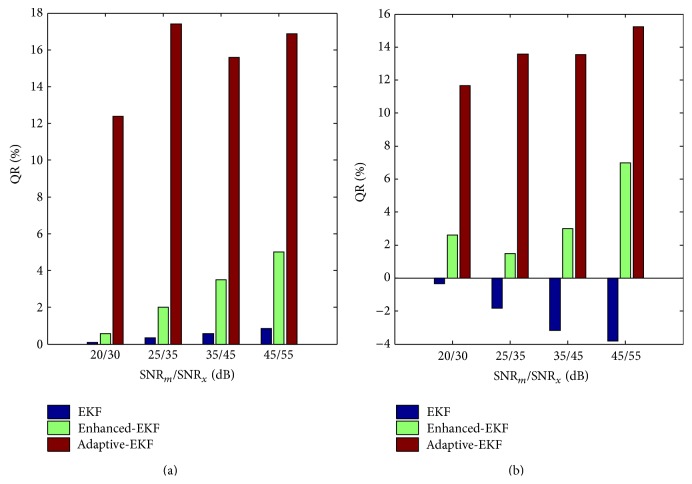
The estimated QRs of *K*_*pe*_ (a) and *K*_*ep*_ (b) with different measurement noise levels.

Next, the robustness of the filters to the measurement noise is investigated with a fixed high SNR_*ω*_ of 40 dB but varying levels of the measurement noise, that is, Cases 4–6 in Table 2. This time the variance of the observation noise, *R*, in the filtering process is set to 3 × 10^−4^ *C*(0), 5 × 10^−4^ *C*(0), 8 × 10^−4^ *C*(0), and 3 × 10^−3^ *C*(0) for Cases 3–6, respectively. The estimated *K*_*pe*_- and *K*_*ep*_-QRs are shown in Figure 7, from which it is again found that the much higher QRs are achieved by the adaptive-EKF as compared to the other two schemes.

#### 3.1.3. Independence to Initialization

**Figure 8:**
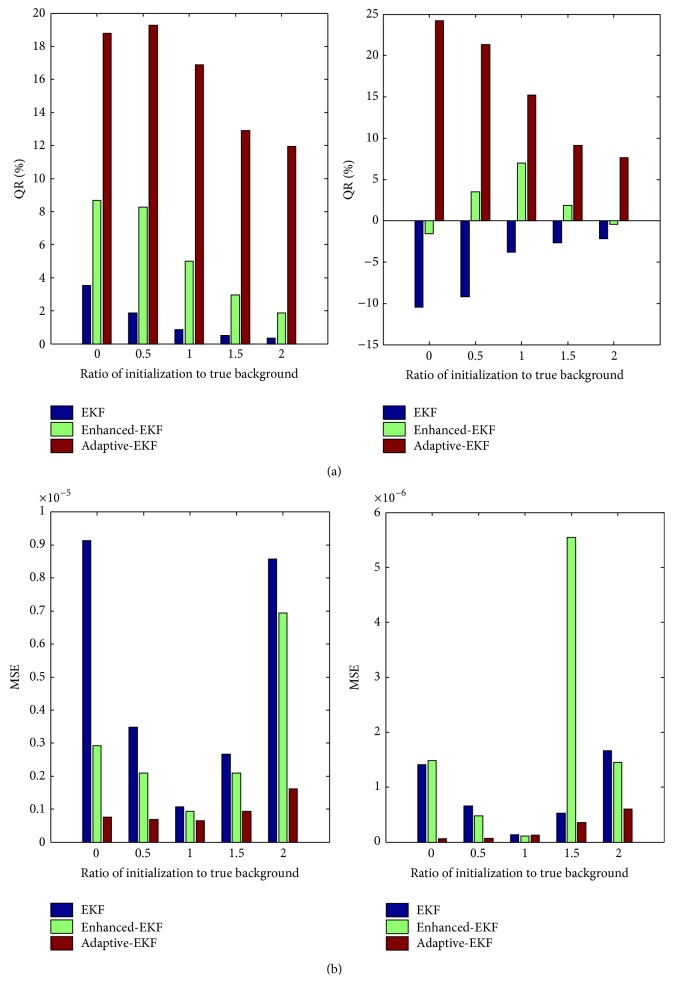
(a) QRs and (b) MSEs of the estimated *K*_*pe*_ (left) and *K*_*ep*_ (right) as the initial *K*_*pe*_ and *K*_*ep*_ are deviated from their true backgrounds with a varying proportional factor from 0 to 2.

The independence of the initialization of *K*_*pe*_ and *K*_*ep*_ is quantified by both the QR and the MSE for the pharmacokinetic contrast of 2, with the same noise levels as in Case 3. The MSE measure is introduced to assess the bias of the estimation and calculated in the whole domain. The variance of the observation noise in the filters is set to *R* = 3 × 10^−4^ *C*(0) for all the cases. The initial pharmacokinetic-rates, *K*_*pe*_ and *K*_*ep*_, are identically set for the three filters, in proportion to the true background ones with a varying factor from 0 to 2. The two measures are illustrated in Figure 8, from which it is clear that the adaptive-EKF achieves significantly larger QR and smaller MSE than the others regardless of the deviations of the initial *K*_*pe*_ and *K*_*ep*_ from their true backgrounds.

### 3.2. 3D Digital Mouse Model

**Figure 9:**
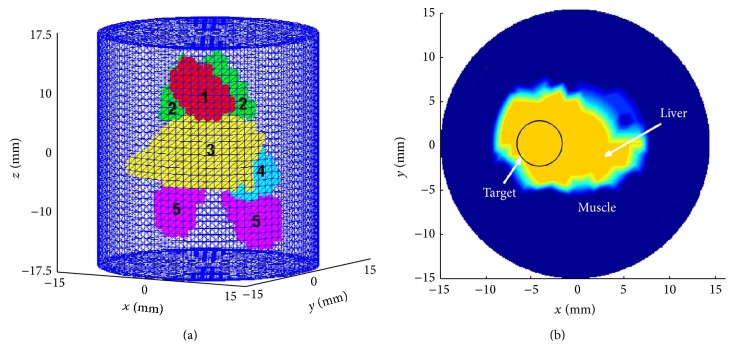
The simple digital mouse model: (a) 3D sketch: 1—heart, 2—lungs, 3—liver, 4—stomach, and 5—kidneys and (b) top view at *z* = 0 plane.

**Figure 10:**
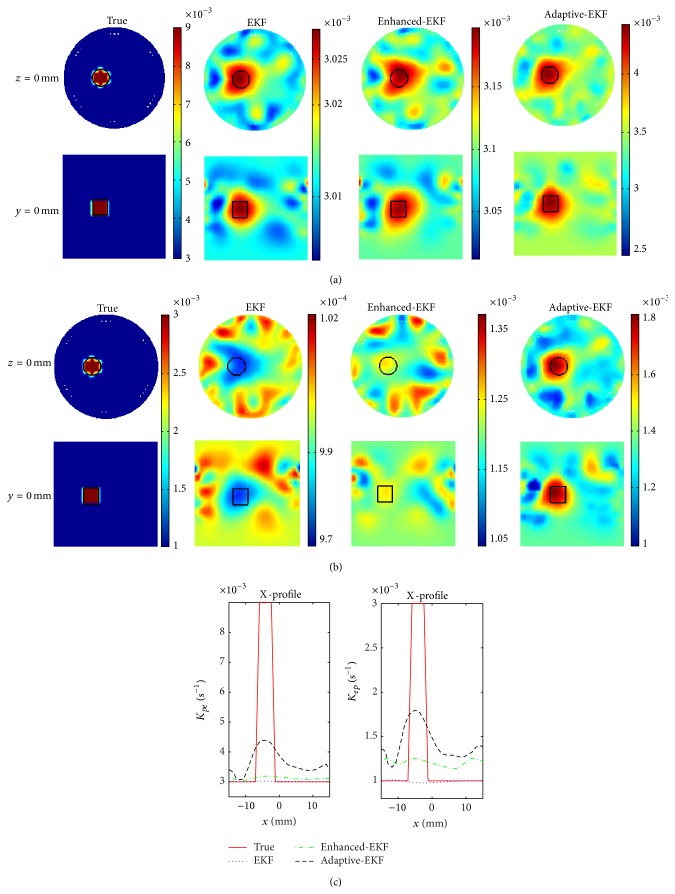
Estimations of pharmacokinetic-rates on 3D digital mouse: (a) the estimated *K*_*pe*_-images at *z* = 0 mm (top view) and *y* = 0 mm (side view), (b) the estimated *K*_*ep*_-images at *z* = 0 mm (top view) and *y* = 0 mm (side view), and (c) the X-profiles of the *K*_*pe*_ and *K*_*ep*_ estimations.

To evaluate the performance of the proposed adaptive-EKF in small animal scenarios, simulated data is generated using the two-compartment model, on a 3D digital mouse atlas (Digimouse) [29]. To facilitate the forward calculation, the mouse model is assumed to be embedded into a cylindrical chamber of 15 mm radius and a 35 mm height filled with the matching fluid, as shown in Figure 9. The cylindrical domain is discretized into 25956 nodes and 47250 elements (prisms) for use with the finite element method. A cylindrical tumor target of 2.5 mm radius and of 6 mm length is placed in the liver with its center at *x* = −4 mm, *y* = 0 mm, and *z* = 0 mm, as shown in Figure 9(b). Five imaging planes along the height (*z*-axis) at *z* = −8 mm, −4 mm, 0 mm, 4 mm, and 8 mm are arranged for data acquisition, with each installing 32 coaxial source-detector optodes around the phantom at equal spacing. The optical parameters of the organs in the digital mouse are listed in Table 3, including the absorption and the reduced scattering coefficients, *μ*_*aυ*_ and *μ*_*sυ*_′, at both the excitation (*υ* = *x*) and emission (*υ* = *m*) wavelengths [30]. The background and target pharmacokinetic-rates are set to those for the case of contrast = 2 in Table 1. The levels of the various noises are set according to SNR_*ω*_ = 30 dB, SNR_*ς*_ = 60 dB, SNR_*x*_ = 45 dB, and SNR_*m*_ = 35 dB. In the filtering process, the variance of the observation noise is chosen as *R* = 8 × 10^−4^ *C*(0), and the initial pharmacokinetic-rates are identically set for the three filters, that is, *K*_*pe*_ = 0.003 s^−1^ and *K*_*ep*_ = 0.001 s^−1^. The top- and side-view images of the estimated pharmacokinetic-rates are shown at *z* = 0 mm and *y* = 0 mm in Figure 10, respectively. Analogous to the 2D scenarios, the proposed adaptive-EKF greatly improves the quantitativeness of the pharmacokinetic estimations as compared to the conventional- and enhanced-EKF, in terms of the X-profiles (*z* = 0 mm and *y* = 0 mm). The results indicate the prospects of the proposed method in ICG kinetics study of diseased mouse models* in vivo*.

## 4. Discussions and Conclusions

It is clearly seen from the estimated pharmacokinetic-rate images that the target sizes are slightly overestimated and biased, even with the adaptive-EKF. These two defects and also the quantitativeness remain to be further improved. In practice, a successful imaging of the pharmacokinetic-rates by the explicit methods, such as the EKFs described here, is dependent on two crucial factors: one is the fidelity of the DFT reconstruction, that is, how to reconstruct the ICG concentration *C*′(*k*) that approaches the realistic one *C*(*k*) and another is the effectiveness of the EKF process, that is, how to accurately extract the pharmacokinetic parameters, *K*_*pe*_ and *K*_*ep*_, from the DFT-reconstructed concentration *C*′.

**Figure 11:**
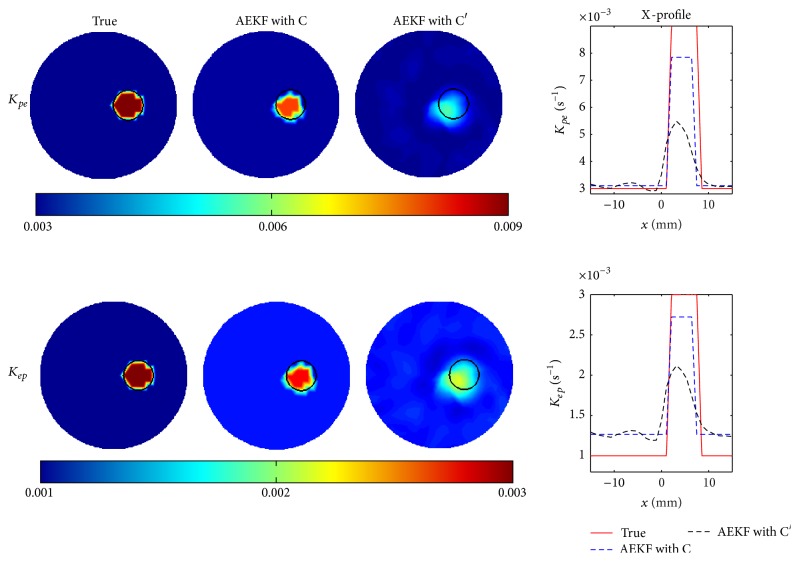
The estimated images of pharmacokinetic-rates from the model-simulated and the DFT-reconstructed ICG concentration, *C* and *C*′, for the case of contrast = 2 in Table 1 with the SNRs set to SNR_*ω*_ = 40 dB, SNR_*ς*_ = 60 dB, SNR_*x*_ = 55 dB, and SNR_*m*_ = 45 dB.

The former is restrained by the severe ill-posedness of the DFT inversion, normally resulting in a decreasing quantitativeness with the increasing contrast [25–27]. The distorted time-course of ICG concentration *C*′ inevitably leads to a deviation in the estimation of the pharmacokinetic-rates. To demonstrate the first effect, Figure 11 compares the estimated images and X-profiles of the pharmacokinetic-rates by the adaptive-EKF from the model-simulated and DFT-reconstructed ICG concentrations, that is, *C* and *C*′, respectively, for the case of contrast = 2 in Table 1 with a SNR setting of SNR_*ω*_ = 40 dB, SNR_*ς*_ = 60 dB, SNR_*x*_ = 55 dB, and SNR_*m*_ = 45 dB. It is obvious that the accuracy of the estimation is significantly improved with the model-simulated concentration *C* by bypassing the DFT process. Nevertheless, the DFT process is indispensable due to the inaccessibility of the ICG concentration in tissue. Therefore, the introduction of more advanced DFT reconstruction methodologies, for example, a prior knowledge guided scheme or an efficient explicit implementation, is necessarily requested for enhancement.

The latter significantly depends on the selection of the noise covariances, that is, **Q**, **Z**, and *R*, in the EKF process. In practice, these noise covariances vary and are unavailable during the measurement period and are suboptimally assumed to be constant in this work. Although the adaptive-EKF can compensate this inaccurate assumption, the prior knowledge or an adaptive updating of the noise covariance may further improve the performance of the estimation.

In conclusion, an adaptive-EKF is developed based on the two-compartment model, for the enhanced estimation of the pharmacokinetic-rates from the dynamic DFT reconstruction. With introduction of a variable forgetting-factor, the propose scheme can effectively compensate the uncertainties of the initial states and the noise covariances. The simulation results suggest that the adaptive-EKF can obtain preferable pharmacokinetic-rate images than the conventional-EKF and the enhanced-EKF with an improved quantitiveness, noise robustness, and initialization independence. The mouse experiments* in vivo* are necessary to study the real pharmacokinetic process in our future work.

## Figures and Tables

**Table 1:** Pharmacokinetic-rates with three contrasts for simulation.

	Target/Background [s^−1^]		
	Contrast = 3	Contrast = 2	Contrast = 1
*K*_*pe*_	0.012/0.003	0.009/0.003	0.006/0.003
*K*_*ep*_	0.004/0.001	0.003/0.001	0.002/0.001
*K*_*p*_	0.025/0.025		

**Table 2:** SNR setting for the noise robustness evaluation.

Case	SNR_*ω*_ [dB]	SNR_*x*_ [dB]	SNR_*m*_ [dB]
1	20	55	45
2	30	55	45
3	40	55	45
4	40	45	35
5	40	35	25
6	40	30	20

**Table 3:** Optical properties of organs in digital mouse.

Tissue	*μ*_*ax*_ [mm^−1^]	*μ*_*sx*_′ [mm^−1^]	*μ*_*am*_ [mm^−1^]	*μ*_*sm*_′ [mm^−1^]
Muscle	0.0052	1.08	0.0068	1.03
Heart	0.0083	1.01	0.0104	0.99
Lungs	0.0133	1.97	0.0203	1.95
Liver	0.0329	0.70	0.0176	0.65
Kidneys	0.0660	2.25	0.0380	2.02
Stomach	0.0114	1.74	0.0070	1.36
